# African swine fever virus DNA is present in non-biting flies collected from outbreak farms in Romania

**DOI:** 10.1186/s13071-024-06346-x

**Published:** 2024-06-28

**Authors:** Oana Maria Balmoș, Angela Monica Ionică, Cintia Horvath, Alexandru Supeanu, Monica Moțiu, Beatris Corina Ancuceanu, Paula Tamba, Florica Bărbuceanu, Vlad Cotuțiu, Mircea Coroian, Sofie Dhollander, Andrei Daniel Mihalca

**Affiliations:** 1 Department of Parasitology and Parasitic Diseases, Faculty of Veterinary Medicine, University of Agricultural Sciences and Veterinary Medicine of Cluj-Napoca, Calea Mănăștur 3-5, 400372 Cluj-Napoca-Napoca, Romania; 2 National Sanitary Veterinary and Food Safety Authority, Piața Presei Libere 1, Corp D1, Sector 1, 013701 Bucharest, Romania; 3 Institute for Diagnosis and Animal Health, Strada Dr. Staicovici 63, Sector 5, 050557 Bucharest, Romania; 4 Clinical Hospital of Infectious Diseases of Cluj-Napoca, Strada Iuliu Moldovan 23, 400348 Cluj-Napoca-Napoca, Romania; 5 European Food Safety Authority, Via Carlo Magno 1A, 43126 Parma, Italy; 6 Faculty of Veterinary Medicine, University of Agricultural Sciences and Veterinary Medicine, Splaiul Independentei 105, Bucharest, 050097 Romania

**Keywords:** ASFV, DNA, Virus, Real-time PCR, CT values, Domestic pigs, Mechanical transmission, Insects, Romania

## Abstract

**Background:**

African swine fever (ASF) is a highly contagious and severe haemorrhagic disease of Suidae, with mortalities that approach 100 percent. Several studies suggested the potential implication of non-biting dipterans in the spread of ASFV in pig farms due to the identification of the ASFV DNA. However, to our knowledge, no study has evaluated the viral DNA load in non-biting dipterans collected in outbreak farms and no risk factors have been analysed. In this context, our study aimed to analyse the risk factors associated with the presence of non-biting dipterans collected from ASF outbreaks in relation to the presence and load of viral DNA.

**Methods:**

Backyard farms (BF), type A farms (TAF), and commercial farms (CF), were targeted for sampling in 2020. In 2021, no BF were sampled. Each farm was sampled only once. The identification of the collected flies to family, genus, or species level was performed based on morphological characteristics using specific keys and descriptions. Pools were made prior to DNA extraction. All extracted DNA was tested for the presence of the ASFV using a real-time PCR protocol. For this study, we considered every sample with a CT value of 40 as positive. The statistical analysis was performed using Epi Info 7 software (CDC, USA).

**Results:**

All collected non-biting flies belonged to five families: Calliphoridae, Sarcophagidae, Fanniidae, Drosophilidae, and Muscidae. Of the 361 pools, 201 were positive for the presence of ASFV DNA. The obtained CT values of the positive samples ranged from 21.54 to 39.63, with a median value of 33.59 and a mean value of 33.56. Significantly lower CT values (corresponding to higher viral DNA load) were obtained in Sarcophagidae, with a mean value of 32.56; a significantly higher number of positive pools were noticed in August, mean value = 33.12.

**Conclusions:**

Our study brings compelling evidence of the presence of the most common synanthropic flies near domestic pig farms carrying ASFV DNA, highlighting the importance of strengthening the biosecurity measures and protocols for prevention of the insect life cycle and distribution.

**Graphical Abstract:**

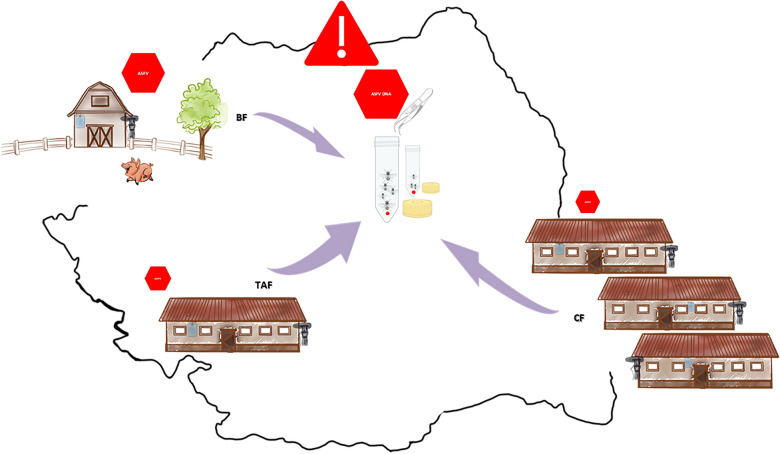

**Supplementary Information:**

The online version contains supplementary material available at 10.1186/s13071-024-06346-x.

## Background

African swine fever (ASF) is a highly contagious and severe haemorrhagic disease of Suidae, with mortalities that approach 100 percent [1]. It is one of the most serious hazards to the pig industry worldwide, including the trade of live animals and pork products [2, 3], being acknowledged as a major transboundary animal disease by the Food and Agriculture Organisation [4].

The ongoing ASF pandemic has had a significant impact on the pig industry worldwide. The disease was reintroduced to Europe in 2007 and has since become endemic in certain regions, causing recurrent outbreaks and economic losses [5]. Since 2017, Romania has experienced the highest number of outbreaks among European Member States [5].

The disease is caused by the ASF virus (ASFV), an enveloped, large, linear, double-stranded DNA virus [6, 7], the only member of the family Asfarviridae [8] and the only known DNA arbovirus [9].

The primary route of introduction of ASFV into farms is direct contact between infected and uninfected pigs or ingestion of contaminated feed [10, 11]. Indirect transmission may occur via various fomites, such as contaminated clothing, surgical equipment, workers, and visitors [12, 13].

Bellini et al. [13] identified seven categories of risk factors for the transmission and introduction of ASFV in domestic pig farms. The listed determinants include biosecurity, swill feeding and slaughtering on the farm, trading of pigs and products, human activity factors and farm management, sociocultural risk factors, ASF in wild boars as a risk for neighbouring farms, and blood-feeding arthropods. The latter have been shown to be able to spread the disease both mechanically (when the vector transfers the agent through the mouth parts or body segments without multiplication) and biologically (when the virus multiplies in the vector) [14, 15]. ASFV's biological transmission was demonstrated only in soft ticks of genus *Ornithodoros*. In Africa, *Ornithodoros moubata* spreads the virus through both the trans-stadial and trans-ovarian pathways. In Europe, ticks of the *Ornithodoros erraticus* complex were implicated in ASFV transmission in Spain and Portugal [1, 16].

Mechanical transmission by haematophagous insects has been incriminated as an alternative route of ASFV transmission, with field studies demonstrating the presence of ASFV DNA in blood-sucking arthropods, such as biting midges, stable flies, mosquitoes, and horse flies [17–21]. Experimental studies have also shown the mechanical transmission to pigs or the persistence of live virus in *Stomoxys calcitrans* [22–24].

In addition, several studies suggested the potential implication of non-biting dipterans in the spread of ASFV in pig farms, based on the identification of viral DNA [18, 19, 21, 25]. Recently, larvae of two commonly found blowfly species, *Lucilia sericata* and *Calliphora vicina*, were experimentally bred on ASFV-infected spleen tissue in a study conducted by Forth et al. [26]. Moreover, larvae of two key insect species produced for food and feed, the mealworm *Tenebrio molitor* and the black soldier fly *Hermetia illucens*, were assessed in experimental exposure studies of insects to ASFV [27].

However, no study so far has evaluated the ASFV DNA load in non-biting dipterans collected in outbreak farms, and no potential risk factors were analysed. In this context, our study aimed to analyse the risk factors associated with the presence of non-biting dipterans collected from ASF outbreaks in relation to the presence and load of viral DNA.

## Methods

**Fig. 1:**
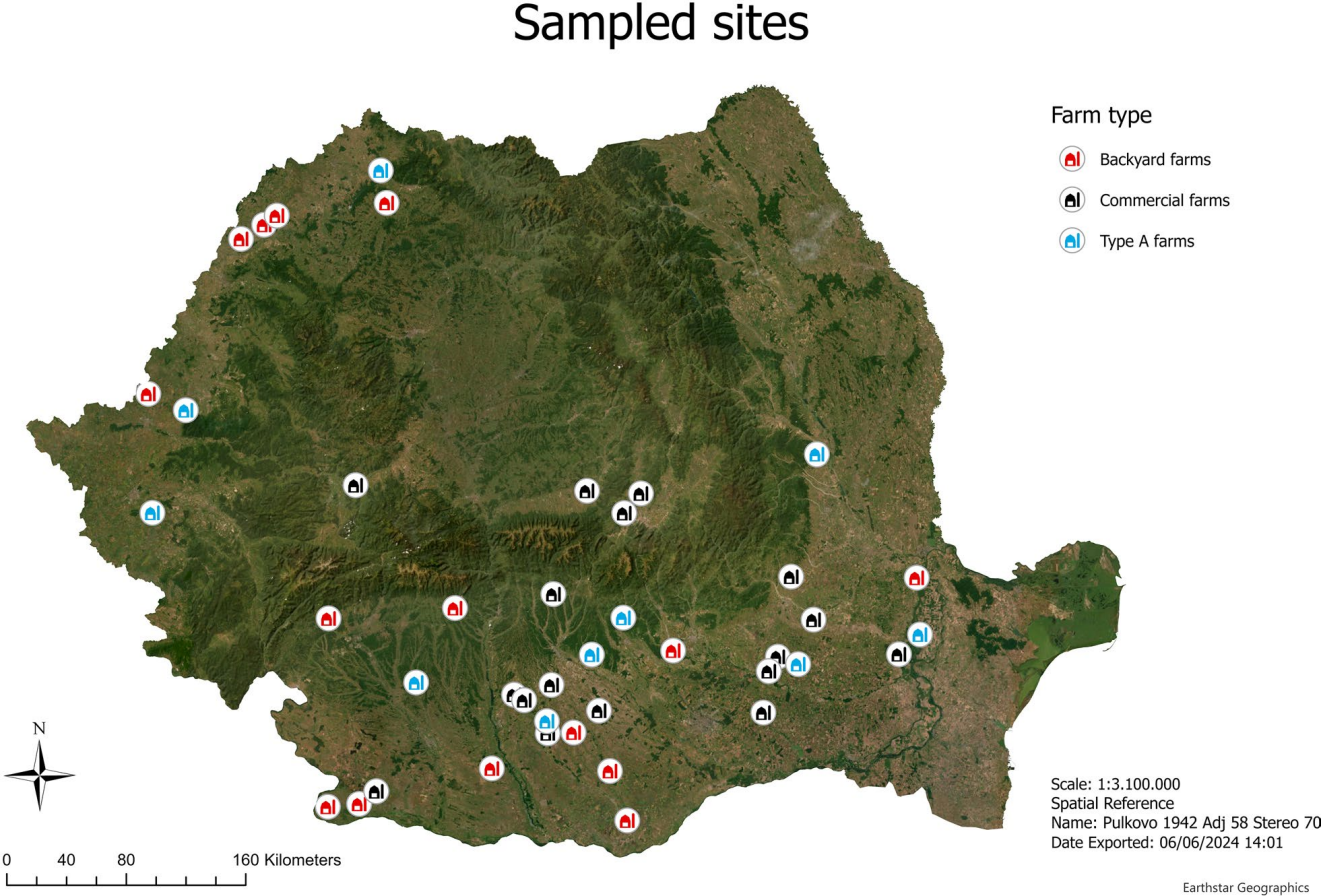
Sampled sites for the insect collection conducted in ASF-positive case farms

Insect trapping was performed in June–September 2020 and August–September 2021 in a total of 42 outbreak farms (corresponding to ASF-positive case farms) (Supplementary file 1: Table S1, Fig. 1). A set of inclusion criteria that prioritized localities (communes) based on a variety of parameters and a scoring system were used for their selection. All the sampling details, protocols, and methodologies are described in Balmoș et al. [20]. Backyard farms (BF) (small, subsistence farms with low levels of biosecurity and production limited to personal consumption), type A farms (TAF) (medium-sized farms with some biosecurity procedures and capable of delivering animals to commercial abattoirs), and commercial farms (CF) (high level of biosecurity and capable of delivering animals to commercial abattoirs) were targeted for sampling in 2020. In 2021, no BF were sampled. Depending on the weather conditions the sampling in each outbreak farm started in maximum 36 h and lasted for 24 h at each site. Each farm was sampled only once, immediately after the ASF outbreak was officially confirmed. The sampling in these farms was carried out for 24 h in each farm.

### Insect collection, morphological identification, and pooling

Non-biting flies, representing by-catch from mini-CDC traps, were separated from biting flies trapped previously [20, 28]. All collected insects were preserved in 70% alcohol. Prior to morphological identification and DNA extraction the insects were kept frozen.

The identification of the collected flies to family, genus, or species level was performed based on morphological characteristics using specific keys and descriptions [29–34].

Pools were made before DNA extraction. Pooling was conducted based on insect group and farm. Due to size differences among groups, large flies (Sarcophagidae, Calliphoridae, Muscidae) were tested in pools of three insects per pool while small flies (Fanniidae, Drosophilidae) in five insects per pool. Pools were only created if the required number of insects per pool (3 or 5) was available. No more than five pools per group and farm were included in the study. The total number of insects included in the study was 1265, divided in 361 pools (Table 1).

**Table 1:** The distribution of the pools during the years

Year	Month				Type of farm			Presence of pigs at sampling		Total
	June	July	August	September	BF	TAF	CF	Yes	No	
2020	87	50	88	58	159	73	51	84	199	283
2021	0	0	54	24	0	14	64	72	6	78
Total	87	50	142	82	159	87	115	156	205	361

*BF* backyard farms, *TAF* type “A” farm, *CF* commercial farm

### DNA extraction and Real-Time PCR

Genomic DNA was extracted using the DNeasy Blood & Tissue kit (Qiagen) according to the manufacturer’s instructions. All DNA samples were stored at −20 °C until processing. All extracted DNA was tested for the presence of the ASFV at the National Reference Laboratory (IDAH-Institute for Diagnosis and Animal Health) using a real-time PCR protocol. Moreover, the extracted DNA was tested according to Standard Operating Procedure (SOP) Identification of the ASFV genome based on the European Union Reference Laboratory for African Swine Fever (ASF EURL) SOPs. This CT (Cycle threshold) value indirectly correlates with the viral load, providing quantitative data for the amount of ASFV DNA. For this study, we considered every sample as being positive with a CT value ≤ 40.

### Statistical analysis and mapping

The statistical analysis was performed using Epi Info 7 software (CDC, USA). The presence of insects and the observed PCR positivity of infection related to each category and its 95% confidence interval (95% CI) were calculated. The differences between categorical variables (insect type, farm type, sampling year, sampling month, presence/absence of pigs) were assessed using Pearson’s chi-square test. The relationship between the mean CT values and categorical variables was assessed using Kruskal-Wallis H test. The odds ratio was established by logistic regression. All results were considered statistically significant at *P* < 0.05.

The correlation between two dichotomous variables, PCR positivity and presence/absence of pigs or sampling year, was assessed by calculation of the phi (Φ) coefficient. The correlation between a dichotomous variable and a categorical one (insect type, farm type, month) was assessed by calculating the point-biserial coefficient. Finally, the association between CT values and categorical variables was evaluated by Spearman’s rank correlation test. The values of the correlation coefficients were interpreted as follows: 0.01–0.20 as none to slight, 0.21–0.40 as fair, 0.41– 0.60 as moderate, 0.61–0.80 as substantial, and 0.81–1.00 as almost perfect.

A multiple regression model was constructed, with the presence of ASFV DNA as the dependent variable, and predictors included sampling year, sampling month, insect genus/species, farm type, and the presence of pigs within the farm as categorical variables.

Mapping of the sites sampled was done using the ArcGIS pro software (version 3.2.2) [35]. The scale used was 1:3.100.000 with the Pulkovo 1942 Adj 1958 Stereo 1970 coordinate system (CRS) (Fig. 1). Furthermore, a spatial autocorrelation (Global Moran’s I) was performed to assess spatial links between the farms sampled and the average CT value. For this purpose, the average CT value for each farm (for CT values > 0) was calculated, along with the average CT value for each insect family/genus/species within each farm (Additional file 2: Dataset S1). Due to sample size limitations the analysis focused on four parameters, total average CT/farm along with *Musca domestica*, Faniidae, and Calliphoridae average CT values/farm.

## Results

### Insect identification

The collected non-biting flies belonged to five families: Calliphoridae, Sarcophagidae, Fanniidae, Drosophilidae, and Muscidae. Flies in Calliphoridae, Sarcophagidae, and Drosophilidae were identified only to family level. For Fanniidae, they were identified to genus level (*Fannia* spp.), while for Muscidae, the insects were identified to species level, with all belonging to two species: *M. domestica* and *Hydrotaea irritans*. In 2020, a total of 283 pools (1027 insects) were prepared and analysed for the presence of ASF virus DNA. The distribution of pools per month of collection and per type of farm is shown in Table 2. In 2021, 14 outbreak farms were sampled, from which 238 insects were included and divided in 78 pools (Table 3).

**Table 2:** Pools of non-biting flies (2020) used for this study

Insects	Month				Type of farm			Presence of pigs at sampling		Total
	June	July	August	September	BF	TAF	CF	Pigs	No pigs	
Calliphoridae	16	5	21	7	21	16	12	18	31	49
Sarcophagidae	5	3	0	1	7	2	0	2	7	9
Drosophilidae	2	0	2	8	10	0	2	4	8	12
*Fannia* spp.	31	14	20	12	50	15	12	15	62	77
*M. domestica*	32	28	45	20	60	45	25	40	85	125
*H. irritans*	1	0	0	10	11	0	0	5	6	11
Total	87	50	88	58	159	73	51	84	199	283

*BF* backyard farms, *TAF* type “A” farm, *CF* commercial farm

**Table 3:** Pools of non-biting flies (2021) used for this study

Insects	Month		Type of farm		Presence of pigs at sampling		Total
	August	September	TAF	CF	Pigs	No pigs	
Calliphoridae	5	2	3	4	6	1	7
Sarcophagidae	4	0	0	4	4	0	4
Drosophilidae	1	0	0	1	1	0	1
*Fannia* spp.	1	0	0	1	1	0	1
*M. domestica*	36	19	10	45	50	5	55
*H. irritans*	1	9	1	9	10	0	10
Total	54	24	14	64	72	6	78

*BF* backyard farms, *TAF* type “A” farm, *CF* commercial farm

### PCR positivity

Of the 361 pools, 201 were positive (55.7%, 95% CI 50.5–60.7) for the presence of ASFV DNA (Table 4). The insect type was statistically significant, with the most positive pools in Calliphoridae and the lowest in *Fannia* spp. The detailed results and their statistical analyses are shown in Additional file 1: Tables S2, S3. There was no correlation between the presence of ASFV DNA and insect group (*r* = 0.004; *P* = 0.93).

**Table 4:** PCR positivity according to sampling year

Year	Pools	Positive	%	95% CI
2020	283	124	43.82	37.95–49.81
2021	78	77	98.72	93.06–99.97

According to farm type, of 361 pools, 159 were collected from BF, 115 from TAF, and 87 from CF (Additional file 1: Tables S4, S6). However, most positive pools were collected in CF (78.3%, 95% CI 69.6–85.1). The results of the statistical analysis, based on the farming system, were found significant: Chi-square test, *Χ*^2^ = 44.81, df = 2; *P* < 0.0001 (Additional file 1: Table S5). A fair correlation was noted between the farm type and PCR positivity (*r* = 0.21, *P* < 0.0001).

According to the presence/absence of pigs at the time of sampling, of the total, positive pools were collected in a higher number when the pigs were still present (71.2%, 95% CI 63.4–78.1) compared with the farms where the pigs were already culled at the time of the sampling, with statistically significant results (Chi-square test, *Χ*^2^ = 25.56, df = 1; *P* < 0.0001) (Additional file 1: Tables S7, S8). A fair correlation was observed (*φ* = 0.27; *P* < 0.0001).

A statistically significant difference (Chi-square test, *Χ*^2^ = 72.47, df = 1; *P* < 0.0001) was also identified between the years of sampling (Additional file 1: Table S9). The difference between the years of sampling and PCR positivity was moderate (*φ* = 0.45; *P* < 0.0001).

There was a statistically significant difference between the overall prevalence of ASFV DNA positive pools according to the month of sampling (Chi-square test, *Χ*^2^ = 32.57, df = 3; *P* < 0.0001), with the highest prevalence in August (74%, CI 95% 65.9–80.9) (Additional file 1: Tables S10, S11, S12).

When the combined effect of multiple predictors was tested by logistic regression, only the presence of pigs and the farm type were retained as significant predictors of ASF DNA positivity (Additional file 1: Table S13).

### CT values

Of the total pools (361), 201 pools were considered positive, with a CT value < 40 (Additional file 1: Table S13). Based on the positivity degree, CT values were considered > 30 weakly positive, 30–24 positive, and < 24 strongly positive. The obtained CT values of the positive samples ranged from 21.54 to 39.63, with a median value of 33.59 and a mean value of 33.56 (Table 5).

**Table 5:** Positivity of pools according to the CT value

PCR results	*n*	%	95% CI
Negative (no CT or CT ≥ 40)	160	44.32	39.28–49.48
Weak positive (30 ≤ CT < 40)	181	50.14	45.01–55.27
Positive (24 ≤ CT < 30)	17	4.71	2.96–7.41
Strong positive (CT < 24)	3	0.83	0.28–2.41

When CT values were assessed by insect group, the overall difference was significant (Kruskal-Wallis test, *H* = 31.22, df = 5, *P* < 0.0001). The lowest mean CT value (corresponding to higher viral DNA load) was observed in Sarcophagidae (Table 6). However, the correlation between insect group and CT values was not significant (*r* = 0.11, *P* = 0.1).

**Table 6:** Distribution of CT values within insect groups

Insect	Min	Max	Median	Mean ± SD
Calliphoridae	21.54	38.27	33.62	33.28 ± 3.07
Sarcophagidae	30.72	34.75	32.52	32.56 ± 1.54
Drosophilidae	30.06	38.61	37.99	35.91 ± 3.61
*Fannia* spp.	32.65	39.63	36.79	36.47 ± 2.02
*M. domestica*	23.32	39.24	33.00	32.97 ± 3.2
*H. irritans*	31.61	39.06	33.12	33.8 ± 2.42

Overall, the difference of mean CT values was significant between farm types (Kruskal-Wallis test, *H* = 38.38, df = 2, *P* < 0.0001), with the lowest being recorded for TAF (Additional file 1: Table S14, S15, S16). There was a fair correlation between farm type and CT value (*r* = 0.38, *P* < 0.0001).

Moreover, the presence of pigs in the farms was associated with a significantly lower mean CT value (Kruskal-Wallis test, *H* = 23.01, df = 1, *P* < 0.0001). A slight correlation was observed (*r* = 0.24, *P* = 0.0004) (Additional file 1: Table S17).

The lowest mean CT values (corresponding to higher ASFV DNA load) were noticed in August. Higher CT values, corresponding to a weaker positivity (> 33.46), were predominant in pools from June, July, and September (Kruskal-Wallis test, *H* = 8.98, df = 3, *P* = 0.029) (Additional file 1: Table S18, S19). A slight correlation was observed (*r* = 0.11, *P* = 0.01).

The multiple regression model indicated the sampling year, insect genus/species, and farm type as significant predictors (*P* < 0.005), (Additional file 1: Table S20).

**Fig. 2:**
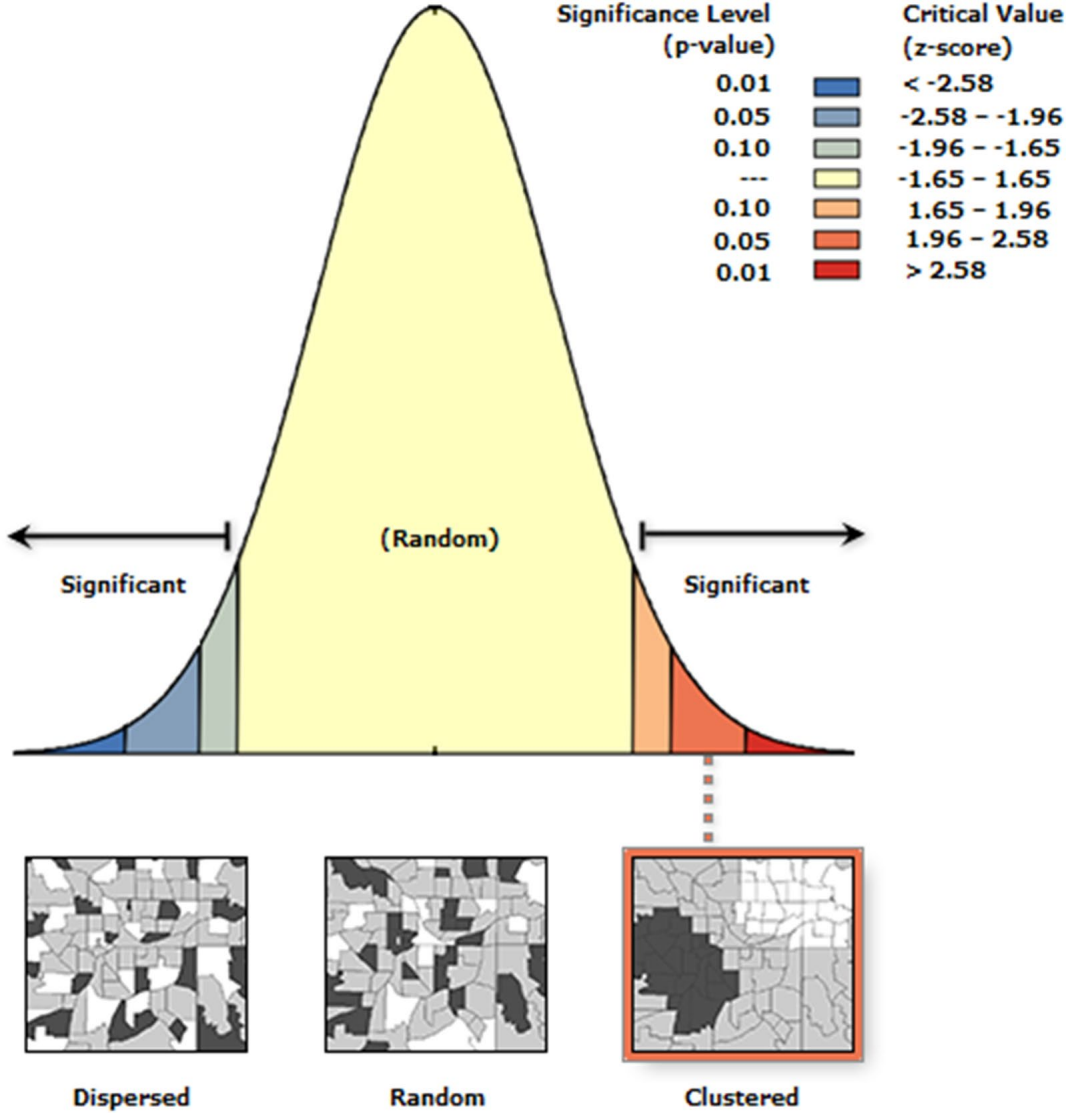
Spatial links between the farms sampled and the average CT value

The spatial autocorrelation test showed a clustered pattern in farms from which *M. domestica* specimens were recovered, with a Moran’s Index of 0.163, *z*-score of 2.159, and *P*-value of 0.03 (Fig. 2). The other parameters investigated yielded a random pattern.

## Discussion

Prior to this study, no previous research had examined the entomological findings from this standpoint. We conducted a thorough analysis and evaluation of the data collected from ASF outbreak domestic pig farms. Our focus was on the quantitative presence of ASFV DNA in numerous specimens. These specimens belonged to various non-biting insect families that are highly significant in the pig farming industry. The investigation took place only during outbreaks of ASF across a span of 2 consecutive years, specifically during the warm period for temperate climates, and covered all three different types of pig farms.

Most of the known fly families are common in livestock farms. These farm insects can represent a significant risk factor in the mechanical spread of infectious diseases. They have access to infected materials, including the carcasses of dead animals, waste, and various fluids and excretions. Transmission of pathogens by adult flies occurs by mechanical dislodgement from their exoskeleton (via pulvilli that are used for adherence to vertical surfaces). These contaminated flies can be harmful in the livestock industry, causing serious outbreaks [36].

Turčinavičienė et al. [21] demonstrated that such synanthropic insects frequently move between the swine farms (mostly over up to 2–3 km), particularly during windy weather, and can therefore act as mechanical vectors for the spread of a variety of diseases.

Due to the availability of manure, pig farms create an ideal setting for the breeding, feeding, and habitation of various fly species. Non-biting flies are involved in the mechanical transmission of economically significant pathogens which affect swine such as porcine reproductive and respiratory syndrome virus, *Streptococcus suis*, *Salmonella* spp., *Escherichia coli*, and classical swine fever [33, 37, 38]. These insects can carry various microorganisms on their pulvilli and exoskeleton and transfer them easily to susceptible hosts. For ASFV, several studies have assessed the presence of viral loads in non-biting dipterans, such as common flies and flesh flies, with both positive and negative results.

Turčinavičienė et al. [21] conducted a study in light of the ASF outbreaks in Lithuanian pig farms. The aim was to provide a large-scale overview of pig-farm-associated Diptera [Muscidae (*Fannia canicularis*, *Hydrotaea dentipes*, *Musca domestica*, *Pyrellia vivida*), Drosophilidae, Calliphoridae (*Pollenia rudis*), Sepsidae (*Sepsis violacea*)], and other unclassified species of Diptera and Arthropoda. The most prevalent family was Muscidae (especially *M. domestica*).

Our study supports these entomological findings regarding the high abundance of *M. domestica* as well as the presence of other families such as Calliphoridae, and Drosophilidae. Moreover, we provide additional evidence supporting the existence of other species in the families Sarcophagidae, Fanniidae, and Muscidae (*H. irritans*) around pig farms in temperate climates.

Turčinavičienė et al. [21] reported the presence of ASFV DNA in Calliphoridae and Muscidae while [18] demonstrated the presence of ASFV DNA in *Musca domestica* (one specimen out of seven) and *Drosophila* spp. (one specimen out of three) collected in outbreaks in Estonia, with high CT (> 38.10), considering CT values up to 40 to be positive.

In our study, of the 361 pools tested, 201 were positive for ASFV DNA, with a lower median value of the CT (33.00) in the case of *M. domestica*. This highlights a higher viral load (corresponding to CT values < 24) in these specimens. Moreover, we obtained the lowest CT values (correlating to higher ASFV DNA loads) in pools belonging to the family Sarcophagidae (median CT value = 32.52).

However, [19] reported no traces of ASFV DNA in a large sample set of arthropods (Diptera: Muscidae, Calliphoridae, Culicidae, Ceratopogonidae, Tipulidae, Scathophagidae, Sarcophagidae, Chironomidae, Psychodidae, Stratiomyidae; Blattaria; Ixodidae; Lepidoptera; Coleoptera; other unclassified arthropods) collected from ASF outbreaks in South Korea.

After analysing the data (in both years) we summarize several of the main findings: (i) the most positive pools to ASFV DNA were found in Calliphoridae, while significantly lower CT values (corresponding to higher viral DNA load) were obtained in Sarcophagidae; (ii) the most positive pools to ASFV DNA were collected in CF, although positive CT values (as categorical data) were significantly more common in TAF; (iii) positive pools were collected in a higher number when the pigs were still present; (iv) highest prevalence was observed in August.

Based on the main findings summarised above, here are our recommendations for understanding the dynamics of ASFV transmission and informing strategies for disease prevention and control: (i) enhanced surveillance by intensifying surveillance efforts, especially during high-risk months such as August, focusing on monitoring insect populations, particularly Calliphoridae and Sarcophagidae, as they show significant associations with ASFV DNA; (ii) temporal monitoring by observing ASFV prevalence over time, identifying seasonal patterns and trends, and allowing for timely interventions and control measures; (ii) targeted fly control measures especially in CF; (iv) farm management practices emphasising the importance of biosecurity measures on farms, especially when pigs are present (even during ASF outbreaks); this may include strict control of pig movements, limiting contact with potentially contaminated environments, and proper disposal of carcasses; (v) public awareness and education by increasing awareness among farmers and the general public about the importance of reporting and responding promptly to ASFV outbreaks as well as educate farmers on best practices to minimise the risk of ASFV transmission, including proper waste management and insect control; (vi) continuing research on insect-ASFV interaction by conducting further studies to understand the mechanisms of ASFV transmission by insects in the field, including the role of different insect species and their behaviours; (vii) adaptive risk assessment by developing a dynamic risk assessment model that considers factors such as pig presence, farm type, and insect populations to predict and prevent potential outbreaks; (viii) collaboration and information sharing by fostering collaboration between veterinary authorities (national and international level), researchers, and farmers to facilitate information exchange and coordination in disease monitoring and control efforts; (x) continued data collection by maintaining and expanding the collection of data on ASFV prevalence, pig populations, and insect dynamics to refine models and strategies over time.

By considering implementing and combining these recommendations, involved stakeholders in porcine health management can contribute to a better understanding of ASFV transmission dynamics and enhance the development of evidence-based strategies for disease prevention and control.

## Conclusions

Overall, our study brings compelling evidence to the presence of most common synanthropic flies close to and inside domestic pig farms carrying ASFV DNA. These findings highlight the factors that influence the spread of ASFV in Romania, outlining the importance of targeted biosecurity measures during insects’ peak periods and at specific types of farms. This emphasises the importance of the strengthening the preventative measures and protocols for the disruption of the insect life cycle and distribution.

### Supplementary Information

Supplementary material 1: Table S1 Number of sampled farms by type and year. Table S2 Overall PCR positivity for ASFV by insect group. Table S3 Comparison of the prevalence of ASFV DNA between insect groups. Table S4 Analysis of insect diversity in various farming systems. Table S5 Comparison of the prevalence of ASFV DNA according to farm. Table S6 Overall PCR positivity for ASFV by farm type. Table S7 PCR positivity for ASFV according to the presence of the pigs. Table S8 Positivity according to the presence of pigs. Table S9 Distribution of pools according to the year of sampling. Table S10 Distribution of pools according to the month of sampling. Table S11 Positivity according to month. Table S12 Comparison of the prevalence of ASFV DNA according to the month of sampling. Table S13 Predictors of ASF DNA positivity. Table S14 Comparison of mean CT values between insect groups. Table S15 Distribution of CT values within farm types. Table S16 Comparison of mean CT values between farm types. Table S17 Distribution of CT values according to the presence or absence. Table S18 Distribution of CT values within the months of sampling. Table S19 Comparison of mean CT values between the months of sampling. Table S20 Multiple regression: year, farm type, and insect category.

Additional file 2: Dataset 1. Average CT value for each farm (for CT values > 0) along with the average CT value for each insect family/genus/species within each farm.

## Data Availability

All data generated or analysed during this study are included in this published article.

## Abbreviations

ASF: African swine fever
ASFV: African swine fever virus
BF: Backyard farms: small, subsistence farms with low levels of biosecurity and production limited to personal consumption
CF: Commercial farms: high level of biosecurity and capable of delivering animals to commercial abattoirs
CT: Cycle threshold
DNA: Deoxyribonucleic acid
EFSA: European Food Safety Authority
EURL: European Union Reference Laboratory
IDAH: Institute for Diagnosis and Animal Health
PCR: Polymerase chain reaction
Real-time PCR: Real-time polymerase chain reaction
SOP: Standard operating procedure
TAF: Type A farms: medium-sized farms with some biosecurity procedures and capable of delivering animals to commercial abattoirs
UASVM: University of Agricultural Sciences and Veterinary Medicine, Cluj-Napoca
