# Supplementary material for: African swine fever virus DNA is present in non-biting flies collected from outbreak farms in Romania
Source: Parasit Vectors. 2024 Jun 28;17:278. doi: 10.1186/s13071-024-06346-x (PMC11212390; doi:10.1186/s13071-024-06346-x)
Supplement: Supplementary file 1 — Supplementary material 1: Table S1 Number of sampled farms by type and year. Table S2 Overall PCR positivity for ASFV by insect group. Table S3 Comparison of the prevalence of ASFV DNA between insect groups. Table S4 Analysis of insect diversity in various farming systems. Table S5 Comparison of the prevalence of ASFV DNA according to farm. Table S6 Overall PCR positivity for ASFV by farm type. Table S7 PCR positivity for ASFV according to the presence of the pigs. Table S8 Positivity according to the presence of pigs. Table S9 Distribution of pools according to the year of sampling. Table S10 Distribution of pools according to the month of sampling. Table S11 Positivity according to month. Table S12 Comparison of the prevalence of ASFV DNA according to the month of sampling. Table S13 Predictors of ASF DNA positivity. Table S14 Comparison of mean CT values between insect groups. Table S15 Distribution of CT values within farm types. Table S16 Comparison of mean CT values between farm types. Table S17 Distribution of CT values according to the presence or absence. Table S18 Distribution of CT values within the months of sampling. Table S19 Comparison of mean CT values between the months of sampling. Table S20 Multiple regression: year, farm type, and insect category. [file 13071_2024_6346_MOESM1_ESM.docx]

**Additional file 1: Table S1** Number of sampled farms by type and year

| Farm type | 2020 | 2021 | Total |
| --- | --- | --- | --- |
| BF | 15 | 0 | 15 |
| TAF | 8 | 2 | 10 |
| CF | 5 | 12 | 17 |
| Total | 28 | 14 | 42 |

BF backyard farms; TAF – type “A” farm; CF – commercial farm.

**Additional file 1: Table S2** Overall PCR positivity for ASFV by insect group

| **Insect** | **Pools** | **Positive (n)** | **Positive (%)** | **95% CI** |
| --- | --- | --- | --- | --- |
| Calliphoridae | 56 | 49 | 87.5 | 75.93-94.82 |
| Sarcophagidae | 13 | 6 | 46.15 | 19.22-74.87 |
| Drosophilidae | 13 | 5 | 38.46 | 13.86-68.42 |
| *Fannia* spp. | 78 | 23 | 29.49 | 19.70-40.89 |
| *M. domestica* | 180 | 106 | 58.89 | 51.33-66.15 |
| *H. irritans* | 21 | 12 | 57.14 | 34.02-78.18 |

**Additional file 1: Table S3** Comparation of the prevalence of ASFV DNA between insect groups

| **Positivity** | ***M. domestica*** | ***H. irritans*** | ***Fannia* spp.** | **Calliphoridae** | **Drosophilidae** |
| --- | --- | --- | --- | --- | --- |
| *M. domestica* | - |  |  |  |  |
| *H. irritans* | X^2^=0, p=1 | - |  |  |  |
| *Fannia* spp. | X^2^= 17.65, p<0.0001 | X^2^= 4.39,  p= 0.02 | - |  |  |
| Calliphoridae | X^2^= 12.87,  p= 0.0001 | X^2^= 6.8,  p= 0.008 | X^2^= 41.82,  p< 0.0001 | - |  |
| Drosophilidae | X^2^= 1.31,  p= 0.24 | X^2^= 0.49,  p= 0.48 | X^2^= 0.1,  p= 0.52 | X^2^= 12.17,  p= 0.0005 | - |
| Sarcophagidae | X^2^= 0.36,  p= 0.39 | X^2^= 0.07,  p= 0.72 | X^2^= 0.76,  p= 0.33 | X^2^= 8.74,  p= 0.002 | X^2^= 0, p= 1 |

**Additional file 1: Table S4** The analysis of insect diversity in various farming systems

| **Insect** | **Pools** | **Farm type** | | | | | |
| --- | --- | --- | --- | --- | --- | --- | --- |
|  |  | **BF** | | **CF** | | **TAF** | |
|  |  | **n** | **%** | **n** | **%** | **n** | **%** |
| Calliphoridae | 56 | 21 | 13.21 | 16 | 13.91 | 19 | 21.84 |
| Sarcophagidae | 13 | 7 | 4.4 | 4 | 3.48 | 2 | 2.3 |
| Drosophilidae | 13 | 10 | 6.29 | 3 | 2.61 | 0 | 0 |
| *Fannia* spp. | 78 | 50 | 31.45 | 13 | 11.3 | 15 | 17.24 |
| *M. domestica* | 180 | 60 | 37.74 | 70 | 60.87 | 50 | 57.47 |
| *H. irritans* | 21 | 11 | 6.92 | 9 | 7.83 | 1 | 1.15 |
| **Total** | 361 | 159 | 100 | 115 | 100 | 87 | 100 |

BF backyard farms; TAF – type “A” farm; CF – commercial farm.

**Additional file 1: Table S6** Overall PCR positivity for ASFV by farm type

| **Farm type** | **Pools** | **Positive (n)** | **Positive (%)** | **95% CI** |
| --- | --- | --- | --- | --- |
| BF | 159 | 60 | 37.74 | 30.18-45.76 |
| CF | 115 | 90 | 78.26 | 69.60-85.14 |
| TAF | 87 | 51 | 58.62 | 47.55-69.08 |

BF backyard farms; TAF – type “A” farm; CF – commercial farm.

**Additional file 1: Table S5** Comparation of the prevalence of ASFV DNA according to farm type

| **Positivity** | **BF** | **CF** |
| --- | --- | --- |
| BF | - | - |
| CF | X^2^= 42.61, p< 0.0001 | - |
| TAF | X^2^= 9.07, p= 0.002 | X^2^= 8.15, p= 0.003 |

BF backyard farms; TAF – type “A” farm; CF – commercial farm.

**Additional file 1: Table S7** PCR positivity for ASFV according to the presence of the pigs

| **Insect** | **Pigs present** | | | | |
| --- | --- | --- | --- | --- | --- |
|  | **yes** | | **no** | | |
|  | **n** | **%** | | **n** | **%** |
| Calliphoridae | 24 | 15.38 | | 32 | 15.61 |
| Sarcophagidae | 6 | 3.85 | | 7 | 3.41 |
| Drosophilidae | 5 | 3.21 | | 8 | 3.9 |
| *Fannia* spp. | 16 | 10.26 | | 62 | 30.24 |
| *M. domestica* | 90 | 57.69 | | 60 | 43.9 |
| *H. irritans* | 15 | 9.62 | | 6 | 2.93 |
| **Total** | 156 | 100 | | 205 | 100 |

**Additional file 1: Table S8** Positivity according to the presence of pigs

| **Pigs present** | **Pools** | **Positive (n)** | **Positive (%)** | **95% CI** |
| --- | --- | --- | --- | --- |
| yes | 156 | 111 | 71.15 | 63.37-78.12 |
| no | 205 | 90 | 43.9 | 37.00-50.99 |

**Additional file 1: Table S9** Distribution of pools according to the year of sampling

| **Insect** | **Year** | | | |
| --- | --- | --- | --- | --- |
|  | **2020** | | **2021** | |
|  | **n** | **%** | **n** | **%** |
| Calliphoridae | 49 | 17.31 | 7 | 8.97 |
| Sarcophagidae | 9 | 3.18 | 4 | 5.13 |
| Drosophilidae | 12 | 4.24 | 1 | 1.28 |
| *Fannia* spp. | 77 | 27.21 | 1 | 1.28 |
| *M. domestica* | 125 | 44.17 | 55 | 70.51 |
| *H.* irritans | 11 | 3.89 | 10 | 12.82 |
| **Total** | 283 | 100 | 78 | 100 |

**Additional file 1: Table S10** The distribution of pools according to the month of sampling

| **Insect** | **Month** | | | | | | | |
| --- | --- | --- | --- | --- | --- | --- | --- | --- |
|  | **June** | | **July** | | **August** | | **September** | |
|  | **n** | **%** | **n** | **%** | **n** | **%** | **n** | **%** |
| Calliphoridae | 6 | 18.39 | 5 | 10 | 26 | 18.31 | 9 | 10.98 |
| Sarcophagidae | 5 | 5.75 | 3 | 6 | 4 | 2.82 | 1 | 1.22 |
| Drosophilidae | 2 | 2.3 | 0 | 0 | 3 | 2.11 | 8 | 9.76 |
| *Fannia* spp. | 31 | 35.63 | 14 | 28 | 21 | 14.79 | 12 | 14.63 |
| *M. domestica* | 32 | 36.78 | 28 | 56 | 81 | 57.04 | 39 | 47.56 |
| *H. irritans* | 1 | 1.15 | 0 | 0 | 7 | 4.93 | 13 | 15.85 |
| **Total** | 87 | 100 | 50 | 100 | 142 | 100 | 82 | 100 |

**Additional file 1: Table S11** Positivity according to month

| **Month** | **Pools** | **Positive** | **%** | **95% CI** |
| --- | --- | --- | --- | --- |
| June | 87 | 39 | 44.83 | 34.15-55.87 |
| July | 50 | 19 | 38 | 24.65-52.83 |
| August | 142 | 105 | 73.94 | 65.92-80.94 |
| September | 82 | 38 | 46.34 | 35.25-57.70 |

**Additional file 1: Table S12** Comparation of the prevalence of ASFV DNA according to the month of sampling

| **Positivity** | June | July | August |
| --- | --- | --- | --- |
| June | - |  |  |
| July | X^2^= 0.35, p= 0.47 | - |  |
| August | X^2^= 18.36, p< 0.0001 | X^2^= 19.34, p< 0.0001 | - |
| September | X^2^= 0.001, p= 0.87 | X^2^= 0.57, p= 0.37 | X^2^= 15.98, p< 0.0001 |

**Additional file 1: Table S13**  Predictors of ASF DNA positivity

| **Predictors** | **Odds ratio** | **95%** | **CI** | **Z** | **p** |
| --- | --- | --- | --- | --- | --- |
| Pigs’ presence | 2.69 | 1.68 | 4.32 | 4.11 | <0.0001 |
| Farm type | 1.64 | 1.25 | 2.17 | 3.51 | 0.0004 |
| Month | 1.09 | 0.89 | 1.35 | 0.83 | 0.4 |
| Insect type | 1.05 | 0.9 | 1.25 | 0.66 | 0.5 |

**Additional file 1:Table S14** Comparison of mean CT values between insect groups

| **CT** | *M. domestica* | *H. irritans* | *Fannia* spp. | Calliphoridae | Drosophilidae |
| --- | --- | --- | --- | --- | --- |
| *M. domestica* | - |  |  |  |  |
| *H. irritans* | H= 0.05; p= 0.82 | - |  |  |  |
| *Fannia* spp. | H= 25.24; p<0.0001 | H= 8.12; p= 0.004 | - |  |  |
| Calliphoridae | H= 1.24; p= 0.26 | H= 0.54; p= 0.46 | H= 18.85; p<0.0001 | - |  |
| Drosophilidae | H= 3.71; p= 0.054 | H= 1.34; p= 0.25 | H= 0.02; p= 0.88 | H= 3.48; p= 0.06 | - |
| Sarcophagidae | H= 0.64; p= 0.52 | H= 0.71; p= 0.4 | H= 10.43; p= 0.001 | H= 1.97; p= 0.16 | H= 2.7; p= 0.1 |

**Additional file 1: Table S15** Distribution of CT values within farm types

| **Farm type** | **Min** | **Max** | **Median** | **Mean ± STDEV** |
| --- | --- | --- | --- | --- |
| BF | 23.32 | 39.06 | 35.64 | 35.25±2.85 |
| CF | 21.54 | 39.24 | 32.95 | 33.02±2.84 |
| TAF | 25.58 | 39.63 | 32.79 | 32.50±3.37 |

BF backyard farms; TAF – type “A” farm; CF – commercial farm.

**Additional file 1: Table S16** Comparison of mean CT values between farm types

| **Farm type** | **BF** | **CF** |
| --- | --- | --- |
| BF | - |  |
| CF | H= 33.73, p< 0.0001 | - |
| TAF | H= 23.57, p< 0.0001 | H= 0.33, p= 0.56 |

BF backyard farms; TAF – type “A” farm; CF – commercial farm.

**Additional file 1: Table S17** Distribution of CT values according to the presence or absence of pigs

| **Pigs present** | **Min** | **Max** | **Median** | **Mean ± STDEV** |
| --- | --- | --- | --- | --- |
| yes | 21.54 | 39.63 | 32.92 | 32.85±2.86 |
| no | 23.32 | 39.06 | 34.59 | 34.43±3.23 |

**Additional file 1: Table S18** Distribution of CT values within the months of sampling

| **Month** | **Min** | **Max** | **Median** | **Mean ± STDEV** |
| --- | --- | --- | --- | --- |
| June | 27.24 | 39.63 | 34.3 | 34.45±3.09 |
| July | 26.37 | 37.63 | 35.02 | 34.31±3.58 |
| August | 21.54 | 39.24 | 33.38 | 33.12±2.94 |
| September | 23.7 | 39.06 | 32.86 | 33.46±3.54 |

**Additional file 1: Table S19** Comparison of mean CT values between the months of sampling

| **CT** | **June** | **July** | **August** |
| --- | --- | --- | --- |
| June | - |  |  |
| July | H= 0.01, p= 0.88 | - |  |
| August | H= 6.46, p= 0.01 | H= 5.64, p= 0.02 | - |
| September | H= 2.35, p= 0.12 | H= 0.96, p= 0.32 | H= 0.06, p= 0.8 |

**Additional file 1:** **Table S20 Multiple regression: year, farm type and insect category**

| **Residuals** | | | | |
| --- | --- | --- | --- | --- |
| **Min** | **1Q** | **Median** | **3Q** | **Max** |
| -1.29875 | -0.38239 | -0.02818 | 0.36958 | 0.74303 |
| **Coefficients** | | | | |
|  | **Estimate** | **Std. Error** | ***t* value** | ***p* value** |
| (Intercept) | -1098 | 137.4 | -7.992 | <0.0001 |
| Year | 0.543 | 0.068 | 7.992 | <0.0001 |
| Insect | 0.061 | 0.012 | 5.016 | <0.0001 |
| Farm type | 0.063 | 0.029 | 2.165 | 0.0311 |
| Pigs present | 0.040 | 0.053 | 0.749 | 0.4546 |
| Month | -0.021 | 0.022 | -0.950 | 0.3430 |

* Residual standard error: 0.4271 on 355 degrees of freedom; Multiple R-squared: 0.2731, Adjusted R-squared: 0.2629 ; F-statistic: 26.68 on 5 and 355 DF, *p* value: < 0.0001
